# Sleep Disturbances, Psychosis Symptoms, and Suicidal Ideation in First Episode Psychosis: An Exploratory Mixed-Methods Study

**DOI:** 10.1093/schizbullopen/sgag001

**Published:** 2026-01-10

**Authors:** Eva Rogers, Mark Gresswell, Simon Durrant, Laura Hancox

**Affiliations:** University of Nottingham, School of Medicine, Mental Health and Clinical Neurosciences, Jubilee Campus, NG8 1BB, United Kingdom; Loughborough University, National Centre for Sport and Exercise Medicine, Epinal Way, LE11 3TU, United Kingdom; University of Lincoln, Department of Psychology, Brayford Wharf, LN57BD, United Kingdom; University of Lincoln, Department of Psychology, Brayford Wharf, LN57BD, United Kingdom; University of Nottingham, School of Medicine, Mental Health and Clinical Neurosciences, Jubilee Campus, NG8 1BB, United Kingdom

**Keywords:** early psychosis, insomnia, self-injury, suicidality

## Abstract

Sleep disturbances are a risk factor for suicidal ideation and are commonly reported amongst individuals experiencing psychosis. Given elevated suicide risk in first episode psychosis (FEP), understanding associations between sleep and suicidality is imperative for informing risk management and intervention. This study explored associations between sleep, psychosis symptoms, and suicidal ideation and the perceptions of those with FEP regarding these experiences. Ten participants experiencing FEP were recruited from Early Intervention services. Participants wore an actigraph for 7 days, completed 3 measures (insomnia severity index, prodromal questionnaire brief, and Beck scale for suicidal ideation), and participated in a semi-structured interview. No significant associations were found between variables, however, descriptive statistics indicated variation in sleep duration, sleep timing, wake after sleep onset, and onset latency. Qualitatively, participants described an extended process of loss, from losing sleep to “losing themselves” and the ability to make safe decisions. Sleep disturbances were considered central to the meaning making of psychosis experiences prior to an acute episode, and as a “trigger” for subsequent experience. Participants discussed reduced emotional control and heightened self-harm or non-suicidal self-injury following sleep disturbances. Participants described a sense of entrapment in their experiences of poor sleep and described suicide as an escape from their current reality. The relationship between sleep and suicidality in FEP warrants further exploration, including understanding of how specific aspects of sleep relate to suicidality, and exploration of the psychological processes in this complex relationship.

## Introduction

Suicide is the leading cause of mortality amongst those with schizophrenia spectrum disorders (SSDs).^1^ Contributing factors to suicide amongst those with SSDs are well explored, and research on risk-factors in earlier phases such as first episode psychosis (FEP), is increasing.^2–5^ First episode psychosis is considered a high-risk period,^6^ as suicide related mortality is highest in the first 5 years of symptom onset.^7^ First episode psychosis suicidal ideation rates range from 26.2% to 56.5%,^8^^,^^9^ and suicide risk is elevated by ~60% compared to those with SSDs.^10^

Researchers have endorsed the need to distinguish between aspects of suicidality (ideation, planning, attempt, and completion) for research specificity, monitoring, and prevention implications.^11^ Much of the current research in FEP includes “suicide risk” as an all-encompassing term, limiting the potential to identify implicated factors. Suicidal ideation offers an important research focus, given it is a consistently reported antecedent of suicide attempts and completion.^3^^,^^12^ To date, much of the literature focuses on suicidal ideation in those with a longer illness duration,^13^ or broader suicide-risk factors in early psychosis.^4^

Sleep disturbances are a known risk factor for suicidal ideation in both clinical^14^ and non-clinical groups.^15^ Sleep disorders, such as insomnia, are associated with 2-fold increased odds of suicidal behavior in those with mental health difficulties.^16^ This association is particularly pertinent for those with SSDs given the greater prevalence of sleep disorders amongst those with SSDs than the general population.^17–20^

Paradigmatic understandings have shifted from disturbed sleep as a consequence of poor mental health towards recognizing that sleep difficulties may play a causal role in the onset and maintenance of psychosis.^21^^,^^22^ Disturbed aspects of sleep (notably timing, variability, duration, and quality) and insomnia are associated with increased positive symptoms, such as delusions, hallucinations, and increased paranoia in SSD’s,^18^^,^^23–25^ and in at risk mental state (ARMS) populations,^26^^,^^27^ which evidenced suggesting sleep disturbance can predict symptom severity at 12 month follow-up in ARMS groups.^28^^,^^29^

Despite associations between sleep and positive symptoms across the psychosis spectrum,^27^^,^^30^^,^^31^ and positive symptoms and suicide in SSDS,^3^^,^^32^^,^^33^ it is unclear whether this association is existent in FEP. Recent longitudinal work found that those experiencing FEP with insomnia were almost 14 times more likely to experience suicidal ideation over the follow-up period than those without, and sleep problems were dose-dependently associated with positive symptoms.^34^ Given that early intervention in FEP is associated with improved prognosis,^35^^,^^36^ this dearth of information regarding sleep as a potential risk factor to symptom exacerbation and functional outcomes such as suicidal ideation is noteworthy.

There are several theories that consider how sleep may be implicated in suicidal processes. Diathesis-stress models are well-cited in understanding psychosis onset^37^^,^^38^and are theoretically key to established models of suicide^39^ whereby sleep deprivation poses a precipitating factor to both ideation and attempt. Additionally, some theories such as the “mind after midnight” hypotheses^40^ consider sleep an explicit contributor to suicidal processes, and proposes excessive nocturnal wakefulness facilitates a period of vulnerability to mechanisms of suicidal behavior in which individuals are more likely to engage in risk-related behavior. Given that when adjusting for the number of people awake in the population at a given time, the risk for suicide is highest at night,^41^ such theories are important to consider for those already holding an elevated risk.

Though preliminary evidence in FEP suggests a similar relationship between sleep, symptoms, and suicidal ideation as shown in SSDs or ARMS, there are limitations in the current evidence. Much of the research has focused on insomnia,^42–45^ often dichotomized by categorically identifying the presence or absence of the disorder rather than exploring distinct sleep parameters; a limitation noted in previous reviews of general population studies.^15^ There is considerable heterogeneity in sleep and suicidality measures, with measures encompassing a singular or binary measure,^13^ which limits exploration of whether specific aspects of sleep relate to suicidality. The current literature is lacking in studies employing actigraphy to explore aspects of sleep, and participant perspectives on this relationship.

Research involving actigraphy in those with SSD’s is limited,^13^ with even less work in early psychosis, which has been highlighted as a long-standing limitation of the literature base.^27^^,^^31^ Actigraphy provides a robust 24-hour picture of sleep and explores how rest-activity patterns may relate to phases of psychosis and other outcomes, such as suicidality.^46^ Further, sleep and suicide are complex,^15^ and qualitative research offers an appropriate method to begin to explore such intertwined processes. However, qualitative methods have been employed sparingly in SSD^47^^,^^48^ and ARMS^49^ groups to explore the sleep-psychosis relationship, with no studies using such methods in FEP. Qualitative work in the broader sleep-suicide relationship is also limited.,^50^ yet there is considerable scope for such methods to explore the complex processes implicated in the sleep-suicide relationship in psychosis.

Relationships between sleep disturbances and suicidality are well-established in the general population,^15^^,^^51^ and longer-term SSD’s,^52^ and evidence for such relationships in FEP is growing.^34^^,^^43^ If sleep disturbances are to be considered beyond being simply a secondary issue or consequence, and instead play a contributory role in the onset and maintenance of psychosis with a proposed relationship to suicidality, further effort is needed to understand such relationships in the early stages of illness to inform screening, prediction, and intervention.

Acknowledging the complexities of this relationship, multi-method approaches would allow identification of how sleep is disturbed, how this disturbance is perceived, and facilitate insight into the psychological processes involved in this relationship. Given limited evidence in this area and the lack of both actigraphy and qualitative measures, this preliminary study aims to contribute to a developing area. Thus, the aims of this study are descriptive. Our primary aim is to descriptively explore actigraphy measured sleep disturbances and the subjective experiences of sleep, psychosis experiences, and suicidal ideation in those with FEP. Secondary aims include:

1. To explore if insomnia or actigraphy-measured sleep disturbances (duration, efficiency, wake after sleep onset [WASO], and onset latency) are related to psychotic symptoms and suicidal ideation in FEP
2. To explore if insomnia is related to psychotic symptoms and suicidal ideation in FEP

## Methods

### Participants

Due to the preliminary and descriptive nature of the study, power calculations were not appropriate to determine sample size. Sample size aimed to be reflective of qualitative recommendations such as “data adequacy” ^53^ to ensure the data provides “meaning-richness” across participant accounts. Recruitment challenges in psychosis services were considered such as low interest, high attrition, risk-related restrictions, and capacity to consent.^54^ Ten participants were recruited from early intervention in psychosis (EIP) services in an NHS Trust. All participants self-reported difficulties with sleep. Inclusion criteria are shown in Table 1. Participants were identified by their responsible clinician and provided consent to participate.

**Table 1:** Inclusion and Exclusion Criteria

*Inclusion*	*Exclusion*
Aged 18-65 years	History of traumatic brain injury or neurological disorders due to altered sleep or sleep disorders^55^^,^^56^
Experiencing FEP (diagnosed by service Psychiatrist). Diagnosis included clinical assessment, medical record review, and liaison with previously involved services.	Substance dependent in the recruitment and/or data collection phase. Alcohol and drug use are associated with altered sleep parameters^57^^,^^58^
Attend early intervention in psychosis services and remain under the care of a responsible clinician	Over 18 years of age. Those <18 may be part of a different participant group (at risk mental state) with different sleep parameters^26^^,^^27^^,^^59^
Have sufficient command of English to undertake interview	Display significant harm to themselves or others, as judged by the responsible clinician
Capacity to provide informed consent, assessed by the responsible clinician and researcher	Experiencing acute episodes whereby they lack capacity to consent

### Procedure

Ethical approval was obtained from an NHS research ethics committee (23/WM/0103) and the research department in the recruiting NHS Trust. A cross-sectional mixed method approach was used combining actigraphy, psychometrics, and semi-structured interviews.

Participants completed 3 psychometric measures and then participated in a semi-structured interview. Participants then wore a wrist-worn Actigraph on their non-dominant wrist for 24 hours a day for 7 days; the length of observation meets actigraphy requirements of >72 hours for a valid measure, and extended monitoring of >5 days reduces measurement error.^60^ Participants were asked to identity their intended sleep time and time of awakening by pressing the event button on the watch face. If not pressed, sleep onset and awakening were identified using the software’s automatic identification algorithm and visually assessed.

### Measures

#### Actigraphy

Sleep disturbances were measured by the CamNtech MotionWatch 8, a wrist-worn triaxial accelerometer, which has been used with individuals experiencing psychosis.^20^ Data were sampled at 30 second epochs. Sleep variables collected include total sleep time (TST), WASO, sleep efficiency (SE), and sleep onset latency (SL) in line with commonly reported variables in ARMS and FEP sleep^27^^,^^31^ and sleep-suicide^15^^,^^61^ research.

#### Psychometrics

Age, gender, and time in service were collected. Participants completed the following measures.

#### *Insomnia Severity Index* *^62^*

The insomnia severity index (ISI) is a 7-item self-report measure to assess the severity and impact of insomnia symptoms over the last 2 weeks. Items are rated on a Likert scale from none (0) to very severe (4) and are summed for a total score. Scores indicate: 0-7 no clinically significant insomnia; 8-14 subthreshold insomnia; 15-21 moderate insomnia; and 22-28 severe insomnia.

#### *Prodromal Questionnaire Brief* *^63^*

The prodromal questionnaire brief (PQB) is a 21 item self-report measure to assess the presence of psychotic symptoms in early psychosis over the past month. The PQB is scored by the number of items endorsed to provide a total score (0-21). The PQB shows good internal consistency^63–65^and has been used in studies targeting FEP referrals.^66^

#### *Beck Suicidal Ideation Scale* *^67^*

The Beck suicidal ideation scale (BSS) is a 21-item measure of suicidal ideation over the past week. Responses are rated on a 3-point Likert scale and items are summed for a total score. Higher scores indicate higher suicidal ideation. The BSS shows high internal consistency and high inter-rater reliability in those with FEP,^68^^,^^69^ and accurately and sensitively detects low and high levels of suicidal ideation.^48^

#### Interviews

Ten individual semi-structured interviews were conducted by the first author (duration range 42-78 minutes) in-person or via telephone at the preference of the participant. The interview explored experiences of sleep, psychosis, and suicidal ideation. An interview guide was developed using previous qualitative literature regarding sleep and suicide^50^ and sleep and psychosis.^49^^,^^70^ The interview schedule was used flexibly with verbal and non-verbal cues to encourage elaboration.

### Statistical Analysis

Actigraphy data was processed via MotionWare software (version 1.4.20, CamNtech, Ltd), and exported into SPSS (version 29) for analysis. Descriptive and frequency statistics were conducted for demographic, ActiGraph, and psychometric variables. Normality assumptions were explored. Histogram boxplots and the Kolmogorov–Smirnov indicated BSS scores D(10) = 0.482, *P* < .001 were significantly non-normal. A two-tailed Spearman’s Rho correlation (*P* < .05) was used to explore bivariate associations due to the small sample size and non-normal distribution between variables.

### Qualitative Analysis

Interviews were audio recorded and transcribed verbatim. Data analysis was guided by Reflexive Thematic Analysis^53^ and the 6-steps of Thematic Analysis^71^ were flexibly and recursively followed throughout the process. Sentence by sentence was coding completed for each transcript, with both semantic (to provide description of participants experience), and latent (to reflect underlying meaning) codes were constructed. The importance of methodological coherence was explicitly considered in the analysis process to ensure rigor.^72^ Thus, multiple coders were not considered appropriate as attempting to reach “consensus” with interpretation and construction of themes was not applicable to the epistemological position.

A Critical Realist epistemological position was adopted to integrate both observable and experiential realities, and each individual “meaning making” of reality exists within and alongside the wider context in which it occurred.^73^ Analysis was primarily inductive and grounded in participants accounts to reflect the latent underlying meaning in the data, however, aspects of analysis were deductive in that knowledge of the broad existing literature (eg, the “mind after midnight hypothesis, and existing research evidencing sleep-suicide relationships”) implicitly provided an “interpretive lens” to orientate data.^74^ The “four R” criterion^72^ and Critical Appraisal Skills Programme^75^ checklist were used to establish rigor.

### Reflexivity

Reflexivity in mixed methods research is considered critical self-awareness, in which researchers attempt to understand how their preconceptions can impact and influence data collection and analysis.^76^ The lead researcher worked as a trainee clinical psychologist, and the research team consisted of 2 clinical psychologists and an academic with a clinical research focus. It was acknowledged that the research team held a psychological perspective of the development and maintenance of psychosis and considered the contribution of sleep in this context. Supervision and research meetings were used to discuss interviews and subsequent themes, with the goal not to reach consensus but offer challenge in interpretation.^72^

## Results

Participant average age was 38.5 years (range 24-62). The average time spent in EIP was 18 months (range 10-29). See Table 2 for participant demographics.

**Table 2:** Participant Demographics

Variable	Mean (range)
Age (years)	38.5 (24-62)
Gender	
*Male*	3
*Female*	7
Currently using sleep aids	10
*Zopiclone*	9
*Promethazine*	1
Time in service (months)	18.9 (10-29)

### Quantitative

There were no statistically significant relationships between actigraphy-measured sleep disturbances (TST, SE, WASO, and SL) and psychotic symptoms and suicidal ideation. There were also no significant relationships between insomnia and psychotic symptoms (r = .43, *P* < .22) or insomnia and suicidal ideation (r = −.62, *P* < .06), although the high correlation coefficients are strongly indicative. Descriptive statistics and correlation coefficients between sleep, symptom, and suicide are presented in Table 3.

About 7/7 nights data were collected for all participants. Mean TST was 6.32 hours and minutes (range 3.57-8.02, SD = 1.04), mean SE was 80.72 (%) (range 49.79-97.28, SD = 14.16%), mean WASO was 65.15 minutes (range 11.14-162.69, SD = 42.73) and mean sleep latency was 27.12 minutes (range 0-88, SD = 28.40). The mean ISI score was 15.1 (range 11-23, SD = 3.87). The mean PQB was 8.8 (range 0-20, SD = 7.29), and the mean BSS 1.3 (range 0-7, SD = 2.75).

There was considerable variation in sleep onset time over the 7 day period ranging between 22:25 and 05:05. Given the small sample size, there was considerable variation between participant sleep and psychometric variables. Individual Actigraphic sleep variables and psychometrics are presented in Table 4.

**Table 3:** Descriptive Statistics and Correlation Coefficients for Sleep, Symptom, and Suicide Measures

	M	SD	Range	1	2	3	4	5	6	7
1. ISI total	15.1	3.87	11-23	**–**	.425	−.621	.541	.379	−.242	.342
2. PQB total	8.8	7.29	0-20	.425	**–**	.035	−.091	−.323	.598	.280
3. BSS total	1.3	2.75	0-7	−.621	0.35	**–**	−.528	−.130	.112	.147
4. Avg TST	6.32	1.04	3.57-8.02	.541	−.091	−.528	**–**	.733	−.418	−.770
5. Avg SE	80.72	14.16	49.79-97.28	−.242	−.323	−.130	.733	**–**	−.758^*^	−.976^**^
6. Avg SL	27.12	28.40	0-88	−.242	.598	.112	−.418	−.758^*^	**–**	.685
7. Avg WASO	65.15	42.73	11.14-162.69	.342	.280	.147	−.770	−.976^**^	.685	**–**

^*^Significant at *P* < .05; ^**^Significant *P* < .01.

Abbreviations: SE, average sleep efficiency percentage (%); SL, average sleep onset latency in minutes; TST, average total sleep time in hours and minutes; WASO, average wake after sleep onset in minutes.

**Table 4:** Average Individual Participant Scores for Actigraphy and Psychometric Variables

Variable	Participant	1	2	3	4	5	6	7	8	9	10
Sleep onset range		23:34 01:53	22:38 02:53	22:03 00:46	01:34 05:05	22:25 23:19	22:26 00:53	00:52 03:47	02:36 04:02	23:06 02:48	21:11 00:50
Wake time range		06:15 09:32	06:20 12:01	06:59 09:11	07:34 02:09	05:50 08:40	06:51 07:38	08:03 11:38	08:55 10:54	07:06 08:50	07:13 08:06
ISI total		19	11	16	11	15	16	13	11	16	23
PQB total		12	0	14	16	3	0	7	2	14	20
BSS total		0	0	0	6	0	0	0	7	0	0
Avg TST		6.48	6.33	8.03	5.54	6.22	7.04	3.56	5.51	6.13	6.24
Avg SE		97.28	84.69	90.87	64.22	78.27	93.02	49.79	85.79	81.94	81.36
Avg SL		0	10.19	37.50	88.00	11.33	5.00	61.08	6.20	32.78	6.5
Avg WASO		11.14	45.33	42.57	87.33	92.11	25.50	163.09	52.20	58.42	75.55

Abbreviations: BSS, Beck suicidal ideation scale; ISI, insomnia severity index; PQB, prodromal questionnaire brief; SE, sleep efficiency; SL, sleep onset latency; TST, total sleep time; WASO, wake after sleep onset.

### Qualitative Analysis

#### Theme 1: Meaning Making Experiences of Sleep and Psychosis

Subtheme 1) Sleep loss and falling off the cliff-edge

All participants discussed gradual and then acute sleep loss before their first episode of psychosis. In most cases, sleep loss accumulated to several days with no sleep:

*Before I went to hospital, I was hardly sleeping … I was struggling to get to sleep at night, but I was struggling to stay asleep as well, that was every day, every night … that went on for a few months—P4.*

*[Before admission] I wasn’t sleeping at all. I wasn’t sleeping for days, sometimes even a week. When I did sleep, I’d sleep for 2-3 hours and then it would be another few days before I slept again—P7.*

Participants discussed a “tipping point” in which they described accumulating sleep loss extended to losing themselves and “detaching” from the shared reality:

*I had this period where I didn’t sleep at all, I couldn’t close my eyes for one minute … it wasn’t gradual, it was explosive, the next day everything changed … I lost myself in so many ways and I would be asking my family, am I real? Am I here? Can you see me?—P5*

*I wasn’t sleeping, and it was definitely more of a manic sensation … it was a slow burn that quickly and exponentially came to a head … it spiralled quickly, it was like a switch had been flipped—P8.*

Participants discussed reasons they struggled to initiate sleep. Some participants described how the voices they hear can interfere with sleep onset and engaging in conversation with them can delay or prevent sleep:

*Sometimes it’s like a recording [the voices] that plays back really, really fast, it could be of one person saying one thing over and over and over again and faster than you could actually talk … I find myself talking back to them so I don’t get to sleep, or I just lay with my eyes closed talking to them. It can go on all night, sometimes from the minute I get into bed and then I just won’t sleep—P4*

For others, an ongoing process of night-time rumination was common in delaying sleep onset, and was considered to influence the “emotional tone” of thought processes, whereby negative thinking was common:

*I start overthinking things, like your brain wakes up and it starts thinking … past stuff, future stuff, just a lot of thoughts. I’d probably go to bed a bit later because I tend to sit up and worry about things sometimes, or I would be waking up in the small hours of the morning just worrying about it and going over it, replaying it again and again and again—P1*

*because you’re not sleeping your mind just flips to the other side and all the negative thoughts would keep me awake at night, I couldn’t switch off and everything I’d think about was negative … worries about the past, worries about the future—P5*

Subtheme 2) A sleep loss-mania-psychosis “pathway”

All participants described a “pathway” of how the sleep disturbances they experienced contributed to periods of mania. Participants constructed their experiences similarly, in that a blurring of hypervigilance and unusual beliefs became intertwined during periods of mania:

*I wasn’t sleeping well … and then things really escalated and then I wasn’t sleeping at all. My mind was very, very hard to switch off … this went on for several days ... on the go, racing thoughts, being on the move. I couldn’t sit down; I was all over the place … some of my memories are a bit vague from that time but I remember feeling unsafe generally, and then that spiralled– p6*

*I wasn’t sleeping at all, and then I had the manic stage … in my manic state I can find it really hard to sleep because my brain is really active and grandiose … when I went back to sleeping, I got better, but then I stopped sleeping again and it got worse, and I was back to being delusional—P7*

Detachment from reality was discussed as a common sensation in the construction of experience following sleep disturbance prior to psychosis.

*the next morning [after not sleeping all night] I became very suspicious of everybody, everything, even myself. I didn’t even think I was real. I remember saying to my other half that I believe my corpse is somewhere else and you’re going to pull it out one day because I don’t feel real. That was when I couldn’t sleep, I didn’t feel myself at all—P5*

*When I was in hospital, it felt like out of body … I ended up being completely out of it. I wasn’t aware of myself at all, and I felt completely lost like I wasn’t in reality, I felt separate, and I was not in reality at all—P7*

Subtheme 3) Sleep loss and psychosis; a cycle

In the construction of each participant’s experience, poor sleep was considered pivotal in how they “made sense” of psychosis and was considered the “trigger” for all that followed:

*Before I got sectioned, it was nearly two weeks that I didn’t sleep for … I was sleeping less and less and then I wasn’t sleeping. I was getting flashing in my eyes, hearing things, thinking people were in my house—P10*

*I wasn’t sleeping, that’s what started it, the lack of sleep … then all of a sudden, they [unusual experiences] started. I wasn’t sleeping so my mind was not processing things properly. Little things … they became bigger in my head, and it felt like there was no rescue, and yeah, one thing lead to another—P5*

Sleep felt pivotal for how vulnerable participants felt regarding the continuation of unusual experiences following their acute episode. Whilst sleep is not discussed as the sole explanation for their experiences, following FEP, participants continued to attribute changes in their unusual experiences to sleep difficulties:

*When I don’t sleep, that’s one of my triggers for psychosis … when I’m tired, I get hallucinations and stuff … I feel like the more tired my body got, the worse my beliefs. They got stronger but I believed them more too. Sometimes I could shake them off, but the more tired you get, the more vulnerable you get—P10*

*If I don’t sleep well, I hear the voices more and feel the sensations more regularly ... there’s definitely a link between when I am tired and the voices are more active, so if I could get some sleep, they’d die down … when I started not sleeping well, they started to ramp back up again and were much more frequent—P1*

*The voices are worse when I don’t sleep … [when I’ve not slept] the voices will try and make me feel bad, like they want me to retaliate … they accuse me of things, they’re louder and they don’t stop …I feel way better if I’ve slept the next day, but I can tell they’re a lot different when I don’t sleep—P4*

#### Theme 2: Losing Sleep, Losing Control, Losing Myself

Participants discussed experiencing extended periods of time where they were losing control of their ability to internally regulate their emotions and their ability to make safe choices as a perceived consequence of disrupted sleep.

Subtheme 1) Losing and attempting to regain internal control: a cycle

Participants described an absence of control with their sleep which increased emotional distress. In some cases, emotions escalated to an unfamiliar intensity which they struggled to control:

*If I don’t sleep through the night it’s almost like I’ve had cocaine and I’m rushing and I’m desperate to hurt the people who are in my head … I have a lot more feelings about stuff when I don’t sleep. It’s pretty bad, its mainly anger. I get a lot of energy [when I don’t sleep] it’s like a rush—P4*

Alongside sleep loss, some participants described that the dreams they experienced contributed to a reduced sense of emotional control that impacted their ability to differentiate between dreams and the shared reality:

*Sometimes my dreams can be more like nightmares, they can be more fear based, or I can see things that are a projection of my fears and things that I think may happen. At one time, some of those dreams appeared to be too real to just be a dream and I started to believe that those things were factual and carry that with me … there was a time when I couldn’t distinguish between is this a dream, or is this a gut feeling—P9*

The use of external agents to regain control of sleep was commonly described, whereby “whatever works” was used in desperate attempts to regain control, often leading to cycles of poor sleep, substance use, and emotional distress. Distress centered around an inability to fall asleep, thus, participants sought agents to reduce time spent awake:

*When my sleeping problems started, I know it’s bad, but I was drinking to the point of passing out and I’d have eight hours sleep, so I thought, oh! One night I got drunk, passed out, and slept for eight hours and I woke up and looked at the time and thought wow I feel great! And that’s how that started—P10*

For some participants, the reliance on external agents to bring about sleep onset was distressing, and created a cycle of sleep, distress, and dependency:

*That was the main reason I took cannabis, to help me sleep … I relied on these external things to help me sleep and wake me up, it felt completely out of my control … I’d go to sleep distressed at the fact I had to self-medicate to sleep and I’d wake up in the same amount of distress … now I can see it, but at the time those things seemed to be helping with sleep—P8*

Other participants discussed the role of prescribed sleep medication as providing a physical “switch off” from the reality they were experiencing:

*Sleep played a big part in getting me back to me … if I didn’t take those [sleeping] pills, I would have continued on that path of not sleeping … it provided a break. I wasn’t in control of my own mind, my thoughts, what I said, what I did. Having a pill controlling one aspect of me helped me regain control of myself eventually … it plugged me back in to what’s going on around me—P9*

*My quality of sleep had just diminished, and that tiredness got to a fever pitch during my episode … I think that was what was so restorative about staying in hospital, to sleep in a completely different space was really, yeah, restorative. Those acute medications were key … It really helped me put myself back together—P8*

However, participants often held a double-edged view towards sleep medication. They felt it was necessary to help with acute experiences, but felt the sleep gained was unnatural, and expressed concern about becoming reliant on their use:

*[medication] isn’t something I want to be on for a long time … that sleep is a different type of sleep. It knocks you out, but you know you aren’t sleeping. It’s almost like a machine that has been shut down. They just turn you off and when it is time [to wake up] they just turn you back on—P5*

*[medication] is helping me sleep but they make me anxious … the idea that I need pills to fall asleep and I don’t know what will happen in the future if I take less dosage … I feel like if I am taking pills, I will be dependent on them and I don’t like that—P3*

Subtheme 2) Loss of sleep and loss of safety

Participants discussed that following periods of not sleeping, they displayed out of character or dangerous behavior. Participants discussed how themes of loss, inclusive of sleep and self-control, lead to a loss of safety for themselves and others.

*I burnt some papers in my bathroom. I’d started to see things on the paper like a helicopter and a police car opposite my house when I wasn’t sleeping well. And later, when I was sleeping better, I haven’t seen this. I burnt a lot of paper and stuff in my bathtub in that period when I couldn’t sleep well—P3*

When discussing these incidents, participants often reflected a negative judgement on their behavior, and expressed such incidents were hugely “out of character”:

*I went out and was behaving bizarrely. I got stopped by the police because I was trying to walk in the middle of the street, and I thought the drivers were going to stop for me … I’ve walked out in front of traffic, jumped out of moving cars … I wasn’t scared at all, I was still so delusional, but now I think I could have been run over. I could have died—P7*

*Before I got sectioned I was acting out of character, loads of risky things. One night I was just walking around the streets in my pyjamas on my own … another time I wanted my friends’ keys to drive home drunk, I would never do that—P10*

In participants’ accounts of such incidents, it was constructed that their ability to “make sense” of their surroundings and make safe decisions was impaired. In some cases, the positive feelings of disinhibition came at a cost, whereby they felt unable to identify the risks of decisions. Participants commonly used language that reflected that this experience was facilitated by a different, or another person:

*It feels good. I know it’s bad [to do these things], but it feels good to be on top of the world and feel like you can do anything. But it’s also terrible because I’m delusional, I’m not taking care of myself, I can’t keep myself safe—P7*

*It accumulated in intensity and escalated into something more noticeable … I wasn’t sleeping at all because I felt so energised … lack of sleep distorted my idea of reality; it made me think I was invincible. I felt like I wasn’t human, I didn’t need human things like sleep and food, we were no longer tired—p9*

Subtheme 3) Losing control; sleep-related self-harm and self-injury

Participants discussed feeling distressed when unable to sleep and described accumulating frustration when sleep deprivation persisted over several nights, which often lead to self-injurious behavior. However, the meaning attached to subsequent self-harm was located in reduced sleep, rather than an increase in the intensity of emotions:

*Once I didn’t sleep for 3 days and I ended up hitting my head on the wall because I tried and tried [to sleep] … I punched the wardrobe too and broke my fingers. I was really anxious because I couldn’t sleep—P3*

In other examples, participants understood their engagement in self-harm or aggressive behavior as a response to an increase in hearing voices or unusual beliefs at night. Participants discussed experiencing reduced emotional control following periods of sleep deprivation, and feeling more unable to cope with their distress at night:

*When the voices are really loud and I can’t sleep I get so agitated. I think [hitting myself] is just a release of that tension, or headbutting doors and punching walls and things like that … or I burn myself. The voices, they’ll go “burn it” and I’ll go and burn it [skin] … it makes me feel good, it makes me feel better. I’m agitated and they make me burn myself and I feel calmer—P4*

Some participants also reflected on feeling less in control of themselves and their decisions following periods of sleep disturbance, which contributed to the use of substances as self-harm, or at other times suicide attempt:

*That one was serious [overdose]. That was when I was going through my bad sleeping stage and my mood was everywhere. That’s why I did it, I was getting fed up … when I don’t sleep, I just feel less on control of myself, like you’re scared of yourself … just scared of what I was capable of doing—P10*

#### Theme 3: Feeling Trapped: Suicide as an Escape

Subtheme 1) Sleep and suicidal thoughts: losing hope

Ambivalence towards living was discussed as a common experience. Whilst many did not feel actively suicidal when not sleeping, there was a collective sense of not wanting to end their life, but wanting life as it was to end:

*There was a time that I wrote a suicide note and I put it away … but after I slept, I woke up and ripped it to pieces. It wasn’t like I wanted my life to end, it was just in that moment I was so fed up and had enough and was like maybe I don’t want to wake up after all. I don’t want to end my life, but I don’t want to carry on living like this. I want it to end—P5*

*If I’m trying to sleep and they’re continually talking [voices], repeating my name again and again. I just get to the point where I’m like, not that I don’t feel like living, but I don’t want to have to cope with it anymore—P4*

However, some participants discussed feeling actively suicidal when experiencing acute sleep disturbances, and attributed these feelings to the reduction of sleep:

*I have felt suicidal because I couldn’t sleep … I was fed up with this problem, not sleeping … [when I hadn’t slept for 3 days] then I wanted to die. I was so tired; I didn’t know what to do—P3*

Participants reflected that the experience of having suicidal thoughts was worse at night with little distraction and the night ahead of them. They described a loss of hope, and a reduction in their ability to think about an alternative future:

*There is something worse about experiencing intrusive thoughts [of suicide] at night. If I do get embroiled in it I think well, it’s going to be a long night, you know, its long hours when you’re disturbed at night—P6*

*You don’t see that there are better days ahead because when you aren’t sleeping your days become so much longer, and you worry that you can’t see the future. People will tell you there is light at the end of the tunnel, but you can’t see that. You’re frightened it might be like that for a very long time—P5*

Subtheme 2) “Fearful is a little word”: distress and entrapment

Participants reflected that sleep can offer an escape and provide a “switch off” from the shared reality. Being unable to sleep, thus unable to escape, facilitated a sense of entrapment in a cycle of negative thinking:

*[after a period of not sleeping] it was scary because it got to the point where I didn’t want to be here anymore, that’s as bad as it got … what used to be, what I used to be in control of, what used to be positive turned negative. That was my thoughts. Everything just flipped to the negative—P5*

In some cases, participants described a “blurring” of sleep and wakefulness, whereby they felt trapped within the unusual experiences they were having. This blending at times contributed to heightened awareness of danger and death that extended to daytime hours:

*I was seeing things that weren’t there, I was somewhere else. When I did fall asleep as it was only for an hour or something, it didn’t feel like a break from delusions because then I was just dreaming about whatever I am delusional about. And when I’d wake up, I would just carry on in that delusion—P7*

*All my dreams are about dying or danger … I wake up all confused, like I don’t know if it’s real or not and I’ll say that’s my dream telling me the future… my dreams blended together, and it made me more paranoid when I was out because I felt like those things [in my dream] were going to happen to me—P10*

Participants also discussed experiences of feeling trapped within feelings of paranoia after disrupted or absent sleep:

*I was paranoid. Fearful is a little word, I was paranoid. I was paranoid about everything around me, I felt like nothing was real. When I’d not slept, it was 10x worse. I felt like I was being spied on—P5*

*I’d never had beliefs like that before [my first episode] I felt like people were out to get me, very paranoid, very unsafe. I had to sleep with my bedroom light on but I couldn’t switch off and sleep because I was so scared—P10*

For some participants, they viewed suicide as the only option to escape what they were experiencing:

*The word that stuck out to me was escape … suicide felt like the only way out for me at the time. Terror was a really prominent part of my episode and feeling like I was a target … I was obsessive about suicide. It was like two sides of the same coin, like one was suicide and the other was I’m going to be killed in some horrific way … I may as well kill myself to prevent someone else from hurting me—P8*

## Discussion

The primary aim of this study was to offer initial descriptive insights into actigraphy measured sleep disturbances, and explore the subjective experiences of sleep, psychosis experiences, and suicidal ideation in FEP. Both the descriptive actigraphy data and qualitative findings indicated areas that may warrant further exploration. Qualitative findings in this study aim to provide context, meaning, and possible explanatory mechanisms between perceived relationships in the actigraphy data, alongside contributing to the qualitative literature on sleep and suicidality in those experiencing psychosis. Thus, the discussion is written to reflect both the discrete differences and intersect between quantitative and qualitative findings.

The use of actigraphy enabled exploration of distinct sleep parameters. All participants wore the watch for the study duration, indicating that actigraphy is feasible to explore sleep parameters in this population. Whilst non-significant associations were anticipated due to the descriptive nature of this study, there was high variability in participant sleep measures that warrant attention.

Average sleep duration ranged from 3 hours 56 minutes to 8 hours 3 minutes. Shorter sleep duration is associated with suicidal ideation in non-clinical groups.^77–80^ However, much of the work in FEP explores “sleep problems,” often measured as a binary yes/no outcome, or categorized by the presence or absence of insomnia. Research has indicated that sleep problems at baseline are associated with increased odds of suicidal ideation at 24 month follow up (with a dose-dependent relationship) and were a predictor of higher symptomology in FEP. Given associations between sleep disturbance and suicidal ideation in FEP when measured by single-item measures, larger-scale studies exploring actigraphy measured sleep duration and its relationship to suicidal ideation in FEP are warranted.

Although qualitatively participants noted that short sleep duration was common, longer sleep latency or the inability to initiate sleep onset was considered more distressing. In this study, average sleep latency was 27.12 minutes (range 0-88), whilst average WASO was 65.15 minutes (range 11.14-162.29). Increased WASO and sleep latency is shown in those with long-term psychosis,^81^ FEP^82^ and ARMS.^28^ Research exploring sleep latency, WASO, and suicidal ideation is limited, but there is some evidence for associations between longer sleep latency and elevated active suicidal ideation in adults.^51^ There is also evidence that the timing of nocturnal wakefulness is significant.^83^ Wakefulness in the early morning hours,^84^ with some research specifying between 4 and 5 a.m.,^85^ has been associated with next day suicidal ideation in those with depression. However, recent research has suggested that WASO may not uniquely characterize those who experience suicidal ideation from those that do not.^86^ Thus, WASO may only be significant for suicidal ideation prediction if it occurs at specific times, but this warrants further exploration both generally and in FEP. Further, this finding indicates that participants may perceive increased sleep latency more distressing than shorter sleep duration, which warrants further exploration of lived experiences perspectives of sleep disturbances and suicidality.

There was considerable variation in the timing of sleep onset both individually and across participants**.** This mirrored qualitative data, in which participants discussed inconsistency in their ability to initiate a consistent sleep onset time. Sleep variability may be relevant to the relationship with suicidal ideation for several reasons. Firstly, given considerable night-to-night variability, deriving an average sleep onset time across study measurement periods may not be appropriate to explore nuanced insights. Secondly, our findings indicate high variability of sleep timing. It is important to also consider variability and fluctuations in suicidal ideation in this relationship. Suicidal ideation has been shown to emerge and fluctuate across days, hours, and minutes,^87^ thus prompting a more recent interest into proximal predictors of suicidal ideation. Studies using ecological momentary assessment (EMA) (repeated real-time sampling of variables in participants natural environments) to sample daily variability have shown decreased sleep duration and quality predicted next day suicidal ideation, but not efficiency,^88^ whereas a recent study indicated that sleep latency and WASO predicted next day passive and active suicidal ideation, but duration did not.^86^ These inconsistencies in the EMA literature base are expected given it is an emerging method, however, EMA may be particularly useful to explore the sleep-suicide relationship in FEP given day-to-day fluctuations in sleep timing, and increased risk of suicide in this group. Finally, the absence of a consistent sleep schedule may warrant exploration. Variability of sleep timing is a noted longitudinal predictor of suicidal ideation,^89^ alongside the aforementioned research indicating wakefulness in specific time-periods may increase suicidal risk. When adjusting for the number of people awake in the population at a given time, the risk for suicide is highest at night,^41^ particularly between the hours of 2 and 4 a.m.^85^ Given the notability of circadian drift in long-term psychosis,^81^^,^^90^ and delayed sleep onset and WASO in FEP and ARMS groups, being awake at this time may confer additional vulnerability in an already vulnerable group.

Much of the preceding discussion may be understood within the context of the “mind after midnight” hypothesis,^40^ which proposes disrupted sleep and excessive nocturnal wakefulness facilitates a period of vulnerability for risky behavior, such as suicide. The hypothesis proposes within this timeframe, individuals may be more vulnerable to mechanisms of suicidal behavior, such as attentional bias and negative affect. Participants construction of their experiences regarding prolonged latency and increased WASO were not directly explored in our study, however, participants discussed an increase in suicidal ideation and self-injurious behaviors when awake at night. The experience of night-time wakefulness is considered distressing and related to suicidal ideation^91^; factors contributing to this relationship have been suggested, such as hopelessness^92^ and rumination,^93^ but are yet to be explored qualitatively. Further, the process of entrapment, as discussed amongst participants in the present study, is considered a key driver of suicidal ideation and subsequent suicidal behavior in the quantitative literature,^94^ and is a central explanatory mechanism to the integrated motivational-volitional model of suicidal behavior.^95^ Entrapment has been considered as a potential psychological mechanism to explain the relationship between sleep disturbances and suicidal behavior.^50^^,^^96^ Sleep disturbances were proposed to moderate perceptions of internal entrapment amongst participants by offering an “escape” from voice hearing or emotional distress^50^ but contributing to and perpetuating entrapment when sleep deprived. Further research is needed in this area to disentangle experiences of entrapment in relation to sleep disturbances and voice hearing as both discrete and intersecting processes. Qualitative work exploring perceptions and experiences of delayed sleep and nocturnal wakefulness in FEP would also offer important insights into the psychological processes in this complex relationship.

Despite studies endorsing the need for qualitative work in sleep-suicide research,^50^ sleep-related qualitative work in those with psychosis is limited.^48^^,^^97^ In this study, qualitative accounts facilitated exploration of concepts that were not explored in actigraphy analysis. For example, whilst our secondary aims sought to explore the proposed relationship between sleep and suicidal ideation, qualitative data indicated that non-suicidal self-injury (NSSI) following periods of sleep disturbance was more commonly experienced. Sleep plays an adaptive role in emotional processing,^98^ and both short sleep duration and diagnosed sleep disorders such as insomnia are associated with NSSI.^99^ What is noteworthy, is that NSSI and risk-taking were commonly discussed in relation to periods of sleep deprivation, but also periods in which sleep was considered irregular or non-restorative. Little attention has been afforded to sleep variability as a risk factor for NSSI, despite the high prevalence of NSSI amongst those experiencing FEP or SSDs.^100–102^ A recent EMA study indicated that sleep irregularity predicted more intense urges to engage in NSSI than sleep duration^103^; this finding is particularly pertinent in this context, given that NSSI is a common risk factor for future suicidal behavior in those with mental health difficulties.^104^ Thus, assessment of NSSI in EIP services may be warranted, alongside further research in this area**.**

In addition to NSSI, in the construction of their experience participants discussed periods of self-reported mania following periods of sleep deprivation. Pervasive sleep disturbance is a well-established feature of presentations characterized by mania as disturbed sleep can contribute to periods of relapse given its role in affect regulation.^105^ Risk-taking (often characterized by impulsivity and impaired decision making) is a noted feature of both mania and sleep deprivation in acute and long-term presentations.^106^ It is estimated that 5%-20% of EIP caseloads consist of individuals experiencing mania,^107^ and whilst neglected in the literature ~30% of those experiencing FEP experience affective symptoms.^108^ Though research is sparse, evidence indicates that those with FEP and mania symptoms experience more positive psychosis symptoms, and mania can delay remission by over a year in comparison to those experiencing FEP without mania.^109^ Notably, those with longer-term affective psychosis are more likely to be women, and less likely to attempt suicide.^110^^,^^111^ In this study, given the high self-reported experience of mania, and a larger proportion of female participants, this may offer some insight into why suicidality was centered around ideation and self-injurious behavior in this participant group. The experience of mania was considered important in participants construction of their experience of sleep and psychosis, and it may be useful to consider mania as a potential mediator or moderator of the sleep-psychosis relationship in FEP. Or, given a percentage of FEP patients report subsyndromal mania symptoms, considering that some individuals are more vulnerable to mania or psychosis^112^ and sleep disturbance may “trigger” such experiences. Qualitative work could explore construction of participants experiences of mania and other psychological processes in the sleep-psychosis relationship.

Actigraphy data indicated that despite short, all participants gained at least several hours of sleep per night in the present study, However, in the qualitative analysis participants described periods of acute sleep deprivation prior to FEP, which warrant exploration in the context of psychosis and suicidality. The duration of complete sleep deprivation was considered important, which reflects experimental literature showing that the severity of psychosis experiences increases with each night of total deprivation.^21^ However, in much of the sleep deprivation literature, once sleep debt is restored, participants have reported full “recovery.”^21^ What is less clear is why some individuals, such as those participating in this study, continue to experience persistent psychosis symptoms following a period of sleep restoration. Epidemiological studies have evidenced a multitude of factors in the etiology of psychosis vulnerability,^36^ and it may be plausible that sleep deprivation may partially contribute to psychosis onset in individuals with an already increased vulnerability, supporting diathesis models of mental health difficulties.^37^ Sleep restriction research may further endorse this point, given the largest effect sizes for increased paranoia and hallucinations were found in those with more psychotic experiences at baseline.^38^ Diathesis-stress models^113^ are theoretically implicated in well-established models of suicide, for example the Motivational-Volitional model,^39^ in which acute sleep disturbance may be considered a precipitating factor in the volitional stage, though this warrants further exploration.

Secondary aims of this study sought to explore if insomnia (as measured by the ISI) was related to experiences of suicidal ideation or psychosis symptoms. Whilst relationships were not significant, correlation coefficients are strongly indicative of a relationship, which aligns with meta-analyses evidencing insomnia as the strongest predictor of suicidal ideation.^15^ All participants met criteria for subthreshold insomnia on the ISI, with 6 participants meeting criteria for clinical insomnia. In this context, another potential explanation for persistent experiences following sleep restoration is that sleep remains only partially restored in some individuals. Much of the sleep restriction literature employs a total deprivation design,^114^ rather than mimicking insufficient sleep over extended periods as in insomnia. Insomnia has been associated with 3-to-4 fold increased odds of suicidal ideation over 3 month,^43^ and 1-8 year periods^52^ in those with SSD’s, and has also shown moderate to strong effect sizes in FEP.^115^ Research regarding relationships between sleep disturbances and suicidality in FEP is still evolving, with considerable methodological heterogeneity present in the few available studies.^115^ However, the strength of evidence is increasing, and recent findings indicate that those experiencing FEP with persistent sleep difficulties were 13× more likely to experience suicidal ideation at least once over a 24 month period than those without.^34^

The above discussion indicates that FEP research exploring sleep as both a proximal and distal predictor of suicidal ideation is warranted. While the next-day or proximal impact of sleep disturbances has started to be considered by EMA research, there is also a need to consider longitudinal risk of suicidal ideation through the accumulative impact of chronic and persistent sleep disturbance in this group. Clinically, it may be appropriate for EIP services to include sleep assessment in routine outcomes and risk assessments given their relationship to both symptom progression and suicidality. Further, sleep difficulties may be targeted through psychological treatments, for example, cognitive behavioral therapy for insomnia (CBT-I) has shown efficacy for those experiencing psychotic disorders (^116^^,^^117^) and in ARMS groups,^118^ though the therapeutic target is often reduction of positive psychotic symptoms alongside improvement in sleep disturbances.^119^ Very few studies include suicidal ideation as an intervention outcome in CBT-I trials for those with psychosis.^117^ However, studies seeking to reduce suicidal ideation through targeting reduction in insomnia in the general population shows promise.^120^ There is less attention in the literature afforded to how targeting sleep disturbances may be an alternative intervention for reducing NSSI, given participants indicated NSSI could be understood as attempts at emotional regulation, or a collateral consequence of frustration following sleep deprivation. Further research is warranted in this area. Given the complexity of suicidality, particularly in FEP, it is likely that CBT-I is most appropriately delivered as an adjunct therapy to other interventions for those experiencing suicidal ideation.^121^ Currently, addressing sleep difficulties are not yet incorporated into best-practice guidance for FEP, though emerging evidence indicates significant benefit, particularly at the early stages of psychosis.^118^

Study findings should be considered in light of limitations**.** Whilst the small number of participants was appropriate for this mixed-methods exploratory study, no significant associations were found. However, qualitative findings indicate meaningful relationships between sleep and suicidality in FEP with several directions for future work. Thus, future research aiming to identify robust associations between actigraphy-measured sleep variables, psychosis symptoms, and suicidal ideation must be adequately powered. Participants in this study were diverse in age; given research has indicated higher risk age groups for suicide,^122^ this may explain the heterogeneity of quantitative findings. Further, higher rates of suicidal behavior have been consistently reported amongst females experiencing FEP,^6^ which is consistent with the gender-specific pattern of suicidal behavior in the general population.^123^ Given the higher percentage of female participants, and other gender-based differences in FEP^124^ that are beyond the scope of this study to consider, findings should be considered in light of this.

All participants were regularly taking hypnotic and anti-psychotic medication. Though the experience of using hypnotics was discussed by participants, considering the interaction between medication was beyond the scope of this study. In addition to the effects of hypnotics, anti-psychotic medication, such as quetiapine and clozapine, have shown efficacy for improving sleep quality,^125^ thus, it is likely that the strength of association between actigraphy and measures of suicidality in this study may partially reflect this improvement in sleep quality.

Actigraphy cannot provide insight into sleep stages and given associations between reduced Rapid Eye Movement (REM) sleep and emotional control,^126^ and emotional control and suicidal ideation,^127^ it is important to acknowledge that this variable was unexplored.

All psychometric measures were self-report. Whilst the PQB indicated the presence of symptoms, measures such as the Clinical Global Impressions scale (CGI) may be appropriate in future studies to provide a clinically robust measure of symptomology in this population, or the PANSS due to its interviewer administration. Additionally, considerable participant diversity likely contributed to the high variation in BSS measures. Due to the structure and wording of the BSS,^5^ many participants scored 0 which may not be reflective of passive or fluctuating ideation. Measures such as the Suicidal Ideation Attributes Scale may be more appropriate in future work to assess attributes of suicidal thoughts rather than quantify their presence. However, it is acknowledged that there is a need for brief and psychologically robust measures of suicidal ideation developed and validated in populations of those with psychosis.

Due to the cross-sectional design, associations were limited to one point in time and the qualitative aspect retrospective. Longitudinal research, including those employing qualitative methodologies, or designs with control groups may be useful. Further, given developments in EMA capturing momentary shifts in suicidality in response to sleep disturbance, future research may employ such methods to explore proximal risk in FEP, as this holds important implications for services. Finally, it is acknowledged that this study will not reflect sleep-suicide associations in acute FEP given participants had capacity to consent, and the responsible clinician deeming risk as low was a participation requirement.

In conclusion, this is the first mixed-methods study to explore actigraphy-measured sleep disturbances associations with psychosis symptoms and suicidal ideation in FEP. Notwithstanding limitations, findings indicated important insights to consider and inform future research. The inclusion of actigraphy was both pragmatic and effective to explore distinct sleep parameters in FEP groups, in an attempt to negate difficulties with single item or binary measures of sleep employed in previous studies.^13^^,^^34^ Further, qualitative methods facilitated insight into the construction of participants’ experiences and the complex psychological processes in the sleep-suicide relationship, and thus, provides rationale for the inclusion of qualitative research in FEP and SSD populations in this field.

## Conflicts of Interest

None declared.
